# Fire-Resistant and Thermal Stability Properties of Fluorosilicone Adhesives by Incorporation of Surface-Modified Aluminum Trihydrate

**DOI:** 10.3390/polym16172510

**Published:** 2024-09-04

**Authors:** Kyung-Soo Sung, Hye-Won Cho, Dae-Ho Lee, Woonjung Kim, Namil Kim

**Affiliations:** 1 Research & Development Center, Protavic Korea, Daejeon 34326, Republic of Korea; kssung@protavic.co.kr (K.-S.S.); hwcho@protavic.co.kr (H.-W.C.); 2 Department of Chemical Engineering, Hannam University, Daejeon 34054, Republic of Korea; ldh9139@naver.com; 3 Department of Chemistry, Hannam University, Daejeon 34054, Republic of Korea; wjkim@hnu.kr

**Keywords:** fluorosilicone adhesive, aluminum trihydrate, flame retardancy, silane coupling agent, shear strength

## Abstract

Fluorosilicone was combined with aluminum trihydrate (ATH) to induce synergistic flame-retardant and thermal-resistant properties. The surface of ATH was modified with four different silane coupling agents. The flammability and mechanical properties of the fluorosilicone/ATH composites were assessed using an UL94 vertical test and a die shear strength test. The change in shear strength was investigated under aging for 1000 h at −55 °C and 150 °C. Pure fluorosilicone had inherent fire resistance and thus achieved a V-0 rating even at 20 wt.% ATH loading. Upon addition of ATH treated with 3-glycidoxypropyl trimethoxysilane, the composites exhibited the highest shear strength of 3.9 MPa at 23 °C because of the additional crosslinking reaction of fluorosilicone resin with the epoxide functional group of the coupling agent. Regardless of the types of coupling agents, the composites exhibited similar flame retardancy at the same ATH content, with a slight reduction in shear strength at 180 °C and 250 °C. The shear strength of the adhesives gradually decreased with aging time at −55 °C, but increased noticeably from 3.9 MPa to 11.5 MPa when aged at 150 °C due to the occurrence of the additional crosslinking reaction of fluorosilicone.

## 1. Introduction

Fluoropolymers consisting of carbon(C)–fluorine(F) and carbon(C)–carbon(C) bonds in their skeletal structures have several attractive properties such as thermal stability over a wide operating temperature in the range of −200 °C to 260 °C, chemical resistance toward many corrosive acids and bases, and abrasion resistance with a low coefficient of friction [1,2,3,4,5]. The stronger C–C bond associated with –CF_2_– (e.g., $∆H=423.4\;kJ/mol\;for\;C_{2}F_{6}$) compared to the C–C bond with –CH_2_– (e.g., $∆H=368\;kJ/mol\;for\;C_{2}H_{6}$) allows them to withstand high temperatures [6,7,8]. In addition, fluoropolymers are capable of delaying the ignition time upon burning with the suppression of smoke generation [9,10,11,12]. The fluorine atom is not easily combined with surrounding oxygen and thus inhibits fire growth. Due to their intrinsic characteristics, fluoropolymers have received immense interest in automobiles, electronics, information, and communication devices. However, one of the major deficiencies for their potential applications lies in their poor compatibility with most conventional polymers and poor solubility in most organic solvents because of their high melting point and chemical inertness [13,14]. Therefore, it is essential to improve the compatibility of fluoropolymers with inorganic fillers to achieve high performance of composite materials.

The use of flame-retardant materials is a prerequisite to ensure reliability and fire safety of high-powered devices. Flame retardancy of polymers is typically achieved by adding flame retardants. Due to harmful impacts on health and the environment, halogen-free flame retardants (FRs) such as metal hydroxide and inorganic phosphorus intumescent have recently been used in industries. Aluminum trihydrate (ATH) is the most commonly used flame retardant due to its wide availability, non-toxic nature, and good stability. When ATH undergoes combustion, it decomposes and releases water into the gas phase, which subsequently reduces the temperature and oxygen concentration in the air. In addition, a thermally stable ceramic-like protective layer (Al_2_O_3_) formed on the surface inhibits the transfer of heat and gas [15,16,17,18]. The UL 94 V tests have been well established and preferred for flammability tests. For electric mobility and electric circuit applications, polymer composites must meet the stringent flame retardancy test of UL-94 V-0 rating. However, the effectiveness of halogen-free FRs is comparatively low in contrast to that of the halogen-containing FRs. Although flame retardancy can be improved by using smaller particles, a large amount of filling, usually more than 50 wt.%, is still unavoidable. The addition of large amounts of flame retardants inevitably leads to the poor fluidity of mixtures and the deterioration of mechanical properties of the composites. For example, the filled adhesives that are intended to be screen printed or automatically dispensed for surface mounting should have specific required viscosities. Various attempts have been made to enhance efficiency, such as the addition of nano-sized particles and the combined use of other flame retardants [19,20,21,22]. The use of ATH nanoparticles having large surface areas is advantageous to improve the flame retardance at low ATH loading, but the nanoparticles are easily agglomerated. It is of utmost importance to pursue homogeneous distribution of nanoparticles into a matrix.

In this study, we combined fluorosilicone resin with ATH to obtain flame-retardant and thermal-resistant composites. Fluorosilicone is inherently fire-resistant, and therefore its composites with ATH are expected to exhibit excellent flame retardancy at low ATH loading. High temperature resistant adhesives are generally brittle at low temperature, lowering the strength of adhesive joints [23,24]. Fluorosilicone is promising because it is stable over the wide temperature range. The ATH surface was modified using four different coupling agents containing fluorine, vinyl, epoxy, and methyl groups in order to improve the compatibility with fluorosilicone. The effects of surface treatment on the thermal and mechanical properties of the composite were investigated. Fire-resistant performance with respect to ATH loading was evaluated using the UL94 vertical test. The thermal stability of the composites was investigated through monitoring the change in die shear strength over aging time at low (−55 °C) and high (150 °C) temperatures, respectively. The reliability was further proved by comparing the water absorption of the unaged and aged composites when exposed to boiling water.

## 2. Materials and Methods

### 2.1. Materials

Fluorosilicone adhesives consisted of 35.2 wt.% of fluorosilicone elastomer (FS-ER-1100, TOPDA, Fuzhou, China), 27.6 wt.% of vinyl fluorosilicone (FS-8019-1000, TOPDA, Fuzhou, China), 36.0 wt.% fluoro crosslinker (FS-8016, TOPDA, Fuzhou, China), 1.0 wt.% bis(2-ethyl hexyl maleate) (TCI, Tokyo, Japan), and 0.2 wt.% platinum catalyst (C1142A, Johnson Mattey, London, UK). Aluminum trihydrate (SG-25LSA, Sibelco, Antwerpen, Belgium) having an average particle size of 5.0 μm was used as a flame retardant. Four different silane coupling agents possessing fluorine (KBM-7103, ShinEtsu, Tokyo, Japan), vinyl (KBM-1003, ShinEtsu, Tokyo, Japan), epoxy (KBM-403, ShinEtsu, Tokyo, Japan), and methyl (KBM-13, ShinEtsu, Tokyo, Japan) functional moieties were used for surface modification of ATH particles.

### 2.2. Preparation and Modification of ATH Particles

The 1.0 g of a coupling agent was completely dissolved in 200 mL methanol by mechanical stirring and then 5 g of ATH particles was added stepwise to a total weight of 50 g, i.e., every 5 min, to the solution. The mixtures were then stirred thoroughly at room temperature for 3 h and at 60 °C for 2 h for reaction, respectively. The product was filtered and washed with deionized water several times. Silane-treated ATH particles were dried at 90 °C in a convection oven for 24 h. All coupling agents were used without further purification. Homogeneous fluorosilicone/ATH mixtures were prepared by mechanical stirring for 1 min, followed by three-roll mill (EXAKT80E, EXAKT, Norderstedt, Germany). The mixtures were stored in a refrigerator at −40 °C prior to use. The homogeneous mixture containing fluorosilicone elastomer, vinyl fluorosilicone, crosslinker, catalyst, and adhesion promoter was thermally cured at 175 °C for 60 min.

### 2.3. Characterization

The viscosity of the fluorosilicone/ATH mixture was measured using a Brookfield rotational viscometer (DV2T, Brookfield Engineering Labs, Middleboro, MA, USA) at room temperature by varying the spindle rotation speed from 0.1 rpm to 20 rpm. Adhesion strength was investigated through a die shear test (Dage Series 4000, Aylesbury, UK). Fluorosilicone adhesive was applied on a silver(Ag)-plated lead frame, and then a square-shaped silicon die in a dimension of 1.25 mm in length and 350 μm in thickness was attached. After thermal curing, shear strength was determined by measuring the force required to separate the Si die from a lead frame at a moving speed of 100 μm/s. The flame retardance of fluorosilicone/ATH composites was assessed by a UL94 vertical test, where the dimension of the specimen is 127 mm in length × 12.7 mm in width × 3.2 mm in thickness. The rating is assigned according to the flaming time, dripping, and ignition of a cotton placed under the tested specimen. The coefficient of thermal expansion (CTE) of adhesives was estimated from the amount of thermal expansion versus temperature using a TMA instrument (Model 2940, TA Instrument, New Castle, DE, USA). A sample placed on the TMA cell was heated from 20 °C to 150 °C at a heating rate of 10 °C/min. The viscoelastic behavior of the resin and its composites was probed in a sinusoidal tension mode using the Pyris Diamond DMA (Perkin Elmer, Waltham, MA, USA) technique. The cured film (200 mm in length × 6 mm in width × 0.3 mm in thickness) was heated from 25 °C to 280 °C at a heating rate of 5 °C/min under a nitrogen environment. The frequency of the dynamic mechanical measurement was fixed at 1 Hz.

The fracture surface of the composites containing untreated and surface-modified ATH was observed using a scanning electron microscope (SEM) (PHENOM-XL, ThermoFisher, Waltham, MA, USA) equipped with energy dispersive X-ray spectroscopy (EDX). Cured fluorosilicone is easily fractured at room temperature and thus directly analyzed. Before characterization, the specimens were sputtered with silver for 90 s using a sputtering device. An accelerated voltage of 15 kV with a spot size of 4 was employed. Micrographs corresponding to the cross-sectional morphology were captured at various magnifications.

The thermal stability of the composites was investigated through monitoring the change in die shear strength over aging time at −55 °C and 150 °C, respectively. For water absorption measurement, the sample was placed in boiling water for 24 h and then surface moisture was removed using a dry cloth. Water absorption was determined by measuring relative weight change. We conducted the die shear and water absorption tests according to MIL-STD-883 and ASTM D570 standard specifications, respectively [25,26].

## 3. Results

### 3.1. Effect of Surface Modification on Mechanical Properties

**Figure 1:**
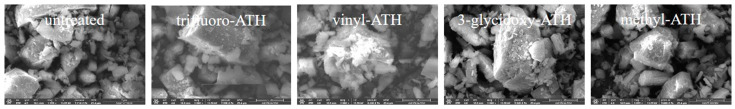
SEM images of untreated and silane-treated ATH surface using trifluoropropyltrimethoxysilane(trifluoro-ATH), vinyltrimethoxysilane(vinyl-ATH), 3-glycidoxypropyl trimethoxysilane(3-glycidoxy-ATH), and methyltrimethoxysilane(methyl-ATH).

To verify the presence of a coupling agent on the surface of aluminum trihydrate (ATH), energy-dispersive X-ray spectroscopy (EDX) analysis was conducted. Table 1 summarizes the atomic and weight percentages for the major elements. As-received ATH (untreated ATH) consists of three main components involving 6.4 wt.% C, 71.8 wt.% O, and 21.8 wt.% Al. The carbon element appears because of the carbon adhesive tape used for sample mounting. These three components are also identified after surface modification. The silicon element from a silane coupling agent is additionally observed after surface modification at the same weight and atomic ratio of 0.1%, indicating that a silane coupling agent is successfully attached onto the ATH surface. In the case of ATH treated with trifluoro-trimethoxysilane (trifluoro-ATH), the fluorine element of 0.2 wt.% and 0.2 at% is detected from -CF_3_ moieties in a coupling agent. Figure 1 displays the SEM images of untreated ATH and silane-treated ATH using four different coupling agents obtained at a magnification of 5000×. No distinct difference in appearance was observed because of low concentration of a coupling agent.

Viscosity is a crucial factor in determining industrial usability. While it is easy to manage the bond line thickness at high viscosity, low viscosity is necessary to achieve a thin-layered uniform coating over a broad area. The viscosity of various fluorosilicone adhesives containing 60 wt.% ATH is tabulated in Table 2. The viscosity of untreated ATH/fluorosilicone (N-ATH) varies from 12,700 to 86,990 cPs depending on the rotation speed (rpm). It was noticed that viscosity increased significantly compared to the fluorosilicone resin observed in a range of 1500~4200 cPs. When the same amount of surface-treated ATH were added, the viscosity increased in the following sequence, i.e., the mixture containing ATH treated with trifluoro-trimethoxysilane (F-ATH), followed by methyltrimethoxysilane (M-ATH), vinyltrimethoxysilane (V-ATH), and 3-glycidoxypropyl trimethoxysilane (G-ATH). As the viscosity of composites are mainly affected by interfacial wettability, the increase in hydrophobicity may contribute to the viscosity. A shear thinning phenomenon in which viscosity decreases with increasing shear speed (rpm) was observed for all the samples. The thixotropic index (TI), the ratio of viscosities generally measured at 0.5 rpm and 5 rpm, was found to be between 2.4 and 3.9. For automatic dispensing purposes in the semiconductor or electronics area, the filled adhesives with a viscosity ranging from 6000 to 20,000 cPs at a rotation speed of 5 rpm and thixotropic index (TI) of 2 to 5 are recommended.

**Figure 2:**
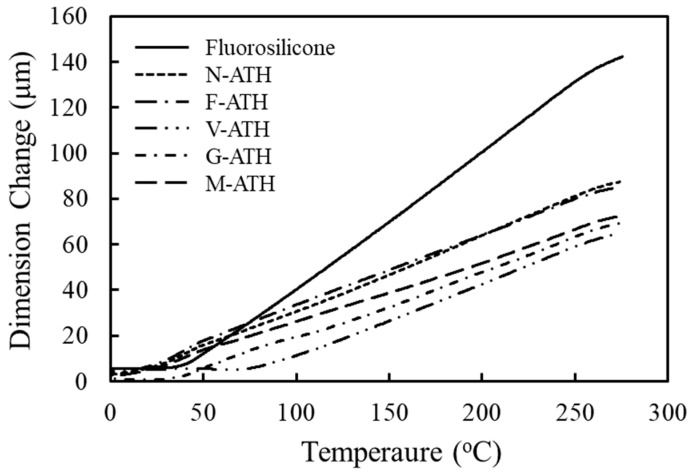
TMA curves of pure fluorosilicone resin and fluorosilicone/ATH composites obtained at a heating rate of 10 °C/min. ATH content of composites was fixed at 60 wt.%.

When the adhesive joint is exposed to temperature changes, the bond experiences significant thermal stress because of the thermal expansion mismatch between the die component and adhesive, causing micro-cracks and damage at the interface. Therefore, the coefficient of thermal expansion (CTE) influences the reliability and life time of adhesives. Figure 2 illustrates the dimensional change of pure fluorosilicone and its composites containing 60 wt.% ATH. Dimensional change increases almost linearly with temperature, but the increase in fluorosilicone resin is more significant in the temperature range from 20 °C to 150 °C. The composites show almost a similar dimensional change in a range of 170~190 ppm/°C regardless of surface modification, which is much lower than pure fluorosilicone of 258 ppm/°C. A lower CTE with the addition of ATH particles makes the composites stiffer, suggestive of an increase in storage modulus. The storage modulus of fluorosilicone/ATH composites measured at 25 °C, 150 °C, and 250 °C is shown in Table 3. As temperature increases, the storage modulus slightly decreases and then increases. Such behavior is associated with the competing effects of softening and additional crosslinking of fluorosilicone at elevated temperature in the presence of Pt catalyst, i.e., between vinyl groups and Si-H groups. The storage modulus of fluorosilicone resin could not be measured because of high stretchability above the glass transition temperature (*T*_g_).

**Figure 3:**
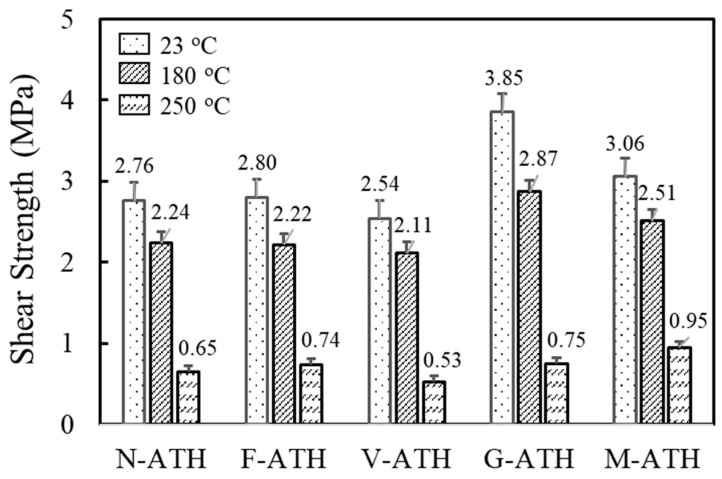
Effects of surface modification on die shear strength between a silicon die and Ag-plated lead frame of fluorosilicone/ATH composites. ATH content of composites was fixed at 60 wt.%.

Figure 3 depicts the temperature-dependent shear strength of fluorosilicone/ATH composites when placed between a silicon (Si) die and silver(Ag)-plated lead frame. Test temperatures of 180 °C and 250 °C were chosen to simulate wire bonding and reflow conditions. Shear strength decreased with increasing measurement temperature due to the combined effects of softening of fluorosilicone and increased difference in thermal expansion between the composites and the Si die. The composite filled with untreated ATH (N-ATH) showed strength values of 2.8 MPa at 23 °C, 2.2 MPa at 180 °C, and 0.7 MPa at 250 °C. The shear strength varied depending on the coupling agents used. The composites treated with 3-glycidoxypropyl trimethoxysilane (G-ATH) exhibited the highest strength for all temperatures measured, i.e., 3.9 MPa at 23 °C, 2.9 MPa at 180 °C, and 0.8 MPa at 250 °C. The epoxide functional group of G-ATH may promote further crosslinking reactions of fluorosilicone and contribute to enhanced shear strength. However, the shear strength values of composites treated with a fluorocarbon silane coupling agent (F-ATH) was not much different from those of untreated ATH, implying that the compatibility effect by introducing a coupling agent was negligible.

**Figure 4:**
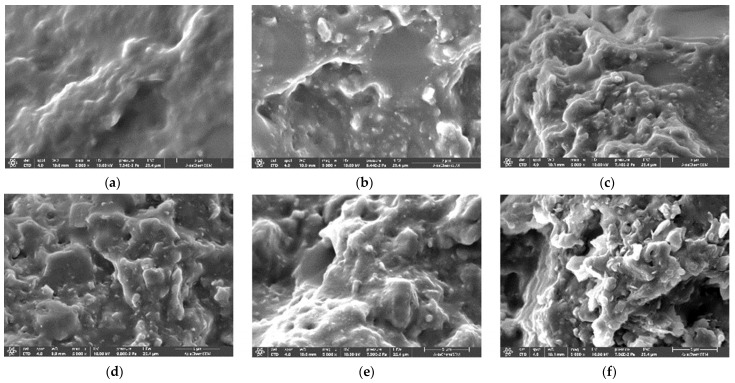
SEM micrographs of fracture surface of pure fluorosilicone and its composites containing 60 wt.% ATH: (**a**) pure fluorosilicone resin; (**b**) untreated(N-ATH); (**c**) F-ATH; (**d**) V-ATH; (**e**) G-ATH; (**f**) M-ATH.

Figure 4 shows the SEM images of the fractured cross-section of the pure fluorosilicone and its composites containing 60 wt.% ATH. In contrast to pure fluorosilicone with a smooth surface, the ATH-filled composites showed an uneven surface with identifiable ATH particles in some areas. As the cross-section was mostly covered with fluorosilicone resin, it was challenging to determine the degree of dispersion and agglomeration of ATH. The observed SEM images suggest that untreated ATH has good compatibility with fluorosilicone. The G-ATH exhibited a broader and smoother matrix region with a small number of ATH particles on the surface due to chemical reaction at the interface between fluorosilicone and a coupling agent on the ATH surface. The morphological investigation implies that improved shear strength was mainly due to enhanced interfacial interaction between fluorosilicone resin and ATH through silane treatment.

### 3.2. Flame Retardancy of Fluorosilicone/ATH Composites

**Figure 5:**
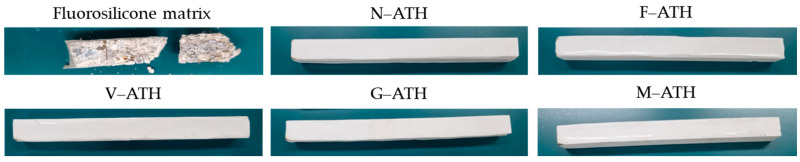
The photographs of the residue of pure fluorosilicone and fluorosilicone/ATH composites modified with different coupling agents taken after the UL94 test. ATH content was fixed at 60 wt.%.

The burning characteristics of pure fluorosilicone resin and fluorosilicone/ATH composites are shown in Table 4. When fluorosilicone resin was exposed to an external flame source, it mostly burned with flame dripping and thus failed to achieve a vertical rating. However, an ignition time of 176 s after the first flame application implies that fluorosilicone is inherently fire-resistant. When 60 wt.% ATH was added, all the composites extinguished immediately after the 1st and 2nd ignition without producing flaming drops, resulting in achieving a UL94 V-0 rating. Figure 5 displays the residue of each composite after the UL94 flammability test. The initial shape was almost retained without any noticeable burning traces except for fluorosilicone resin, indicating that no ignition occurred during the flammability test.

**Figure 6:**
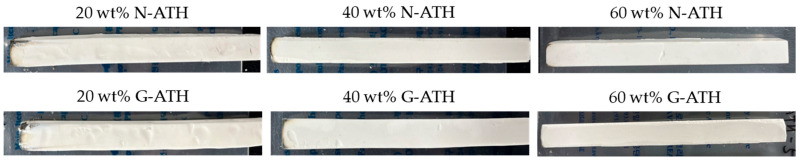
The photographs of fluorosilicone/ATH composites at various ATH content taken after the UL94 test.

Based on the shear strength results of the composites, G-ATH was chosen to investigate the effect of ATH content on flame retardancy. For the purpose of comparison, N-ATH was also used. The flame-retardant behavior of the composites containing 20 wt.%, 40 wt.%, and 60 wt.% ATH are listed in Table 5. At 20 wt.% ATH, burning was not sustained with total burning time of 17 s for N-ATH and 19 s for G-ATH, respectively, which satisfies V-0 rating requirements. Upon increasing ATH content to 40 wt.%, the composites were no longer ignitable after applying a flame without dripping. It is reasonable to infer that the flammability of the composites is not affected by surface modification using a coupling agent. Photographs of fluorosilicone composites having different ATH contents are shown in Figure 6. Partially burning traces were identifiable at the edge of the specimen containing 20 wt.% and 40 wt.% ATH after the flammability test. It can be seen clearly that the composites exhibited better flame-retardant performance as ATH content increased.

### 3.3. Thermal Resistance of Fluorosilicone/ATH Composites

**Figure 7:**
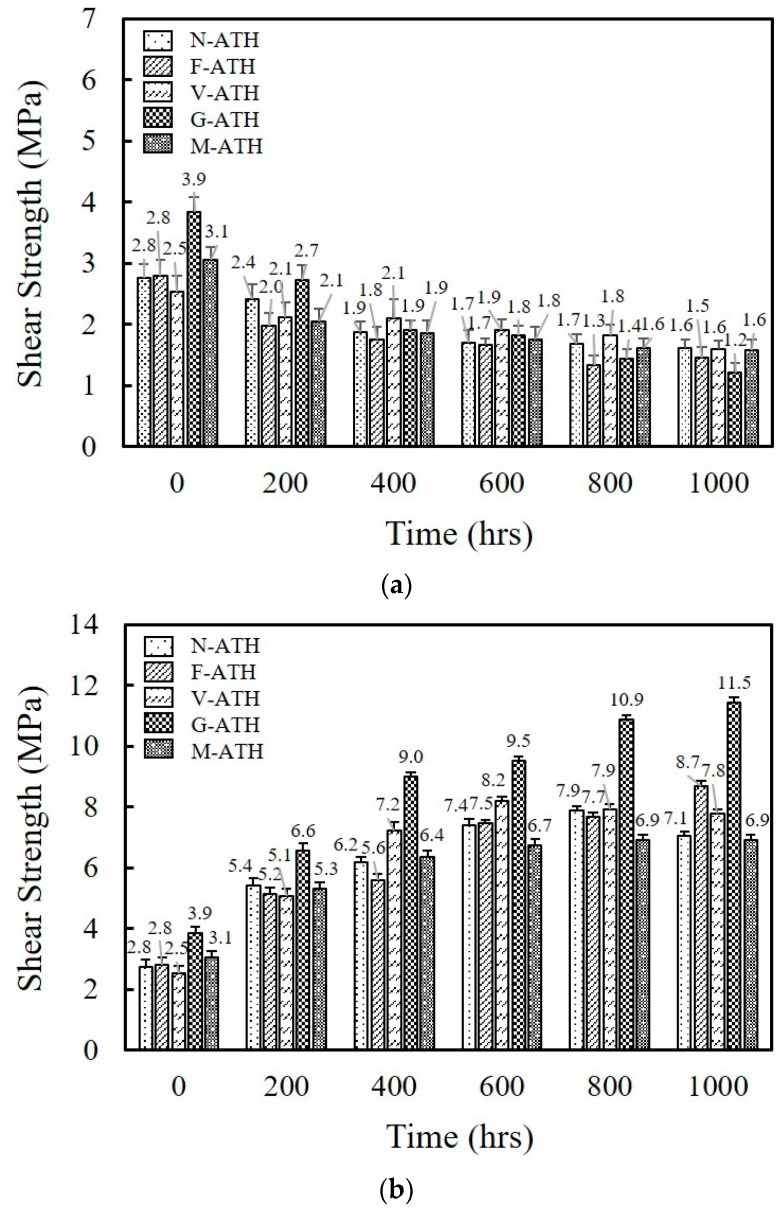
Effects of thermal aging on the shear strength of fluorosilicone/ATH composites at (**a**) −55 °C and (**b**) 150 °C. ATH content of composites was fixed at 60 wt.%.

The long-term reliability of fluorosilicone adhesives was estimated by measuring the shear strength over aging time at −55 °C and 150 °C, respectively. The test conditions were designed following the JESD22 standard [27]. Figure 7a illustrates the variation of shear strength with aging at −55 °C. A gradual decrease was observed with aging time. After aging for 1000 h, the shear strength value decreased by 32.0% for N-ATH, 52.4% for F-ATH, 24.5% for V-ATH, 52.4% for G-ATH, and 39.2% for M-ATH as compared to the strength obtained before aging. The test temperature was still above the glass transition temperature (*T*_g_) of fluorosilicone resin, and therefore a stress generated by the transition to a glassy state could be disregarded. The flexibility of fluorosilicone contributes to the resistance to low temperature.

On the other hand, shear strength increases significantly with aging time for all the composites when subjected to aging at 150 °C (Figure 7b). Despite the expected decrease in shear strength due to thermal expansion mismatch at elevated temperature, the additional crosslinking reaction of the fluorosilicone through post-curing may dominate the adhesion strength. G-ATH maintained higher strength as compared to other composites at all aging times, reaching 11.7 MPa after aging for 1000 h. Aging results clearly indicate that fluorosilicone still maintained a certain level of strength to the bonded substrates, such as a Si die and Ag-plated lead frame.

**Figure 8:**
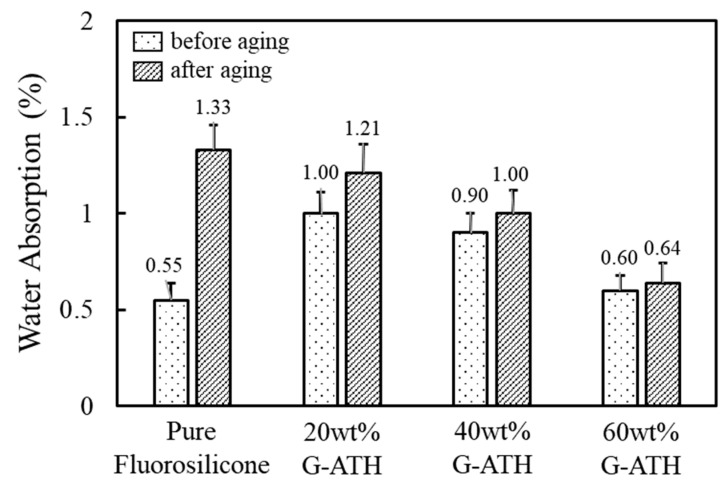
Water absorption of pure fluorosilicone and fluorosilicone/ATH composites treated with 3-glycidoxypropyl trimethoxysilane (G-ATH) before and after aging. The sample was aged at 150 °C for 100 h.

Water absorption of unaged and aged G-ATH are plotted in Figure 8. Although water absorption slightly increased with addition of ATH, the ratio remained below 1.5%. This behavior was closely associated with the intrinsic hydrophobicity and low surface tension of fluorosilicone. Water absorption was relatively high after aging for all compositions. Regarding G-ATH, water absorption gradually decreased as ATH content increased. Enhanced crosslinking density induced by additional reaction between the fluorosilicone and epoxide group of a coupling agent suppresses water absorption.

## 4. Conclusions

The synergistic effects on thermal resistance and flame retardance have been demonstrated by combining fluorosilicone resin with aluminum trihydrate (ATH). The fluorosilicone composites containing 60 wt.% ATH maintained high shear strength at elevated temperatures due to their inherent thermal stability. The addition of ATH decreased shear strength of fluorosilicone adhesives, but this could be recovered by conducting surface modification using a silane coupling agent. The composites treated with 3-glycidoxypropyl trimethoxysilane showed more than 1 MPa higher shear strength as compared to untreated composites at the same composition. Additionally, the fluorosilicone composites reached a V-0 rating at 20 wt.% ATH loading due to the synergistic effects of flame retardant fluorosilicone. After aging at −55 °C for 1000 h, shear strength declined by about 37~68% depending on the type of coupling agent. When aged at 150 °C, the strength increased significantly while maintaining low water absorption due to the occurrence of additional crosslinking. It can be concluded that the combination of fluorosilicone and ATH is effective in inducing synergism in terms of flame retardancy and mechanical properties. Moreover, there is a high demand for fluorosilicone adhesives that can withstand large temperature gradients in electronics, automotive, and aerospace for the purpose of bonding requiring moisture resistance as well as protecting hardware components exposed to fuels.

## Figures and Tables

**Table 1:** EDS analysis of untreated and surface-treated ATH using trifluoropropyltrimethoxysilane(trifluoro-ATH), vinyltrimethoxysilane(vinyl-ATH), 3-glycidoxypropyl trimethoxysilane(3-glycidoxy-ATH), and methyltrimethoxysilane(methyl-ATH).

Element	Untreated ATH		Trifluoro-ATH		Vinyl-ATH		3-Glycidoxy-ATH		Methyl-ATH	
	Atomic (%)	Weight (%)	Atomic (%)	Weight (%)	Atomic (%)	Weight (%)	Atomic (%)	Weight (%)	Atomic (%)	Weight (%)
C	6.4	4.2	6.1	4.1	6.5	4.3	5.2	3.4	8.3	5.6
O	71.8	63.3	72.0	63.4	71.3	62.8	74.1	65.7	71.1	63.4
F	-	-	0.2	0.2	-	-	-	-	-	-
Al	21.8	32.5	21.6	32.2	22.1	32.8	20.6	30.8	20.5	30.9
Si	-	-	0.1	0.1	0.1	0.1	0.1	0.1	0.1	0.1

**Table 2:** Viscosity of fluorosilicone adhesives filled with 60 wt.% ATH modified with different silane coupling agents.

Shear Rate (rpm)	N-ATH	F-ATH	V-ATH	G-ATH	M-ATH
0.1	86,990	252,700	107,700	91,130	149,100
0.5	38,110	72,910	42,250	38,100	53,850
1	26,100	42,670	28,580	27,340	33,970
5	15,660	18,890	16,650	16,240	18,890
10	14,000	15,160	14,500	14,080	16,200
20	12,700	12,800	13,070	12,780	14,310

**Table 3:** Coefficient of thermal expansion (CTE) and storage modulus of pure fluorosilicone and fluorosilicone/ATH composites with variation of temperature. ATH content of composites was fixed at 60 wt.%.

Shear Rate (rpm)	CTE (ppm/°C) 20~150 °C	Storage Modulus (MPa)		
		25 °C	150 °C	250 °C
Fluorosilicone	258	-	-	-
N-ATH	174	110	90	100
F-ATH	189	110	82	94
V-ATH	178	110	80	100
G-ATH	186	110	100	110
M-ATH	178	110	100	110

**Table 4:** The UL-94 results of fluorosilicone and its composites containing 60 wt.% ATH modified with different coupling agents.

Fluorosilicone/ATH	Combustion Time (s)		Dripping	Rating
	After 1st Ignition (t1)	After 2nd Ignition (t2)		
Fluorosilicone	176	burned	D	No rating
N-ATH	0	0	N	V-0
F-ATH	0	0	N	V-0
V-ATH	0	0	N	V-0
G-ATH	0	0	N	V-0
M-ATH	0	0	N	V-0

**Table 5:** The UL-94 results of fluorosilicone adhesives filled with 20 wt.%, 40 wt.%, 60 wt.% of ATH.

ATH Content (wt.%)		Combustion Time (s)		Dripping	Rating
		After 1st Ignition (t1)	After 2nd Ignition (t2)		
N-ATH	20	4	13	N	V-0
	40	0	0	N	V-0
	60	0	0	N	V-0
G-ATH	20	8	11	N	V-0
	40	0	0	N	V-0
	60	0	0	N	V-0

## Data Availability

The raw data supporting the conclusions of this article will be made available by the authors on request.
